# Scattering near-field optical microscopy at 1-nm resolution using ultralow tip oscillation amplitudes

**DOI:** 10.1126/sciadv.adu1415

**Published:** 2025-06-11

**Authors:** Akitoshi Shiotari, Jun Nishida, Adnan Hammud, Fabian Schulz, Martin Wolf, Takashi Kumagai, Melanie Müller

**Affiliations:** ^1^ Department of Physical Chemistry, Fritz-Haber Institute of the Max-Planck Society, Faradayweg 4-6, 14195 Berlin, Germany.; ^2^ Institute for Molecular Science, National Institutes of Natural Sciences, 38 Nishigonaka, Myodaichi-cho, 444-8585 Okazaki, Japan.; ^3^ The Graduate University for Advanced Studies, SOKENDAI, Shonan Village, 240-0193 Hayama, Japan.; ^4^ Department of Inorganic Chemistry, Fritz-Haber Institute of the Max-Planck Society, Faradayweg 4-6, 14195 Berlin, Germany.; ^5^ CIC NanoGUNE, Tolosa Hiribidea 76, 20018 Donostia-San Sebastián, Spain.

## Abstract

Scattering-type scanning near-field optical microscopy (s-SNOM) allows for the observation of the optical response of material surfaces with a resolution far below the diffraction limit. Based on amplitude-modulation atomic force microscopy (AFM) with typical tapping amplitudes of tens of nanometers, a spatial resolution of 10 to 100 nm is routinely achieved in s-SNOM. However, optical imaging and spectroscopy of atomic-scale structures remain a substantial challenge. Here, we developed ultralow tip oscillation amplitude s-SNOM (ULA-SNOM), where the ultraconfined field localized at a 1-nm-scale gap between a plasmonic tip and sample is combined with frequency-modulation (noncontact) AFM in a stable cryogenic ultrahigh vacuum environment. Using a silver tip under visible laser illumination with a constant 1-nm amplitude oscillation, we obtain a material-contrast image of silicon islands on a silver surface with 1-nm lateral resolution, which surpasses the conventional limits of s-SNOM. ULA-SNOM paves the way for the acquisition of optical information from atomic-scale structures, such as single photo-active defects and molecules.

## Teaser

Elastic scattering-light detection meets noncontact atomic force microscopy, reaching a spatial resolution of 1 nm.

## INTRODUCTION

The combination of optical spectroscopy with scanning tunneling microscopy (STM) enables the optical characterization of material surfaces, nanostructures, and molecules as well as their optical control with a resolution far beyond the diffraction limit of light (*1*–*3*). Using plasmonic STM tips, near-field optical techniques in low-temperature (LT) STM (*4*, *5*) have been successfully applied to realize optical spectroscopy at the single- or even submolecular level, including tip-enhanced Raman spectroscopy (*6*, *7*), STM-induced luminescence (*8*, *9*), and tip-enhanced photoluminescence spectroscopy (*10*, *11*). Moreover, the plasmonic near-fields in STM junctions allow for controlling single-molecule photoreactions (*12*–*15*) and visualizing photocurrents through molecular orbitals (*16*). In these works, localized surface plasmon resonances occurring at the nanometer-sized tip apex, so-called nanocavities, are enhanced and confined to a 1-nm^3^-scale volume by the plasmonic coupling between the tip and sample at a 1-nm scale gap (*5*, *17*, *18*). In addition, picocavities, formed by the atomistic structure of the tip apex, can provide further spatial confinement of the plasmonic field inside the narrow gap (*19*, *20*). Such extreme confinement leads to both a localization of the incident light to the atomic scale and a very strong enhancement of optical light emission and scattering from the junction (*5*). Operation at LT and under ultrahigh vacuum (UHV) conditions also facilitates the stable formation of such 1-nm-scale plasmonic gaps.

In parallel to the aforementioned nanocavity/picocavity-based STM studies, scanning near-field optical microscopy (SNOM) has been established as a standard tool for measuring the local dielectric response of materials. The most well-established and sensitive approach is scattering-type SNOM (s-SNOM) (*21*, *22*), where amplitude-modulation atomic force microscopy (AM-AFM), also known as tapping-mode AFM, is used to modulate the localized near-field light at the tapping frequency and then detect the demodulated scattering signal at higher harmonics of the tapping frequency using a lock-in amplifier (*23*, *24*). This scheme eliminates contributions from the far-field background and outputs the part of the scattering signal that strongly depends on the tip-sample gap distance. This method has enabled the visualization of surface plasmon and phonon polaritons (*25*–*28*), phase transitions (*29*, *30*), and individual biological molecules/complexes (*31*, *32*), as well as in probing ultrafast dynamics at the nanoscale (*33*–*35*).

Increasing the spatial resolution to the angstrom scale remains an outstanding challenge in s-SNOM and other scattering-light detection techniques (*36*–*42*). The spatial resolution of conventional s-SNOM is typically limited to tens of nanometers. While this resolution is sufficient for many applications including the observation of polariton wavelengths longer than ~50 nm (*27*), s-SNOM has not yet accessed more localized structures such as single molecules (*43*) and photo-active point defects (*44*). One of the approaches to achieve high-resolution s-SNOM is to detect higher-harmonics signals, giving rise to weak yet strongly localized response. On the basis of this approach, so far 5 or 6 nm resolution was reported as best cases (*32*, *41*, *42*).

As an alternative approach, the use of a sufficiently low amplitude of the cantilever oscillation is expected to be advantageous to sensitively detect light scattering from near-fields localized to the Angstrom scale. The small tapping essentially enhances the duty cycle for sampling of strongly confined structures, substantially enhancing the sensitivity to localized signals from ultranarrow tip-sample gaps. Such an approach was proposed by previous studies on the tapping-amplitude dependence of the s-SNOM images both experimentally (*45*) and theoretically (*46*, *47*). However, AM-AFM inherently requires tapping amplitudes greater than ~10 nm to prevent the tapping tip from adhering to the sample surface. Furthermore, in AM-AFM with low tapping amplitude, the tip motion tends to be anharmonic (*48*–*50*), giving rise to difficulty in interpreting lock-in demodulated signals (*46*). These problems can be overcome by frequency-modulation AFM (FM-AFM) (*51*), also known as noncontact AFM, using a quartz tuning fork (QTF) sensor (*52*) as a cantilever. The stiffness of the cantilever and the constant oscillation-amplitude feedback in the FM mode allow for a stable oscillation with a constant, small amplitude ( A≲1 nm) (*53*). Operation in LT-UHV environments not only leads to high force sensitivity for FM-AFM with high Q values of the oscillation but also stabilizes an ultranarrow tip-sample gap as used in LT-STM. Recently, the advantage of combining optical excitation with hybrid STM/FM-AFM systems has been demonstrated in the low-frequency terahertz regime (*54*).

In this study, we demonstrate FM-AFM–based s-SNOM with an ultralow, 1-nm-scale, cantilever oscillation amplitude, which we refer to as ultralow tip oscillation amplitude s-SNOM (ULA-SNOM). This enables the generation and detection of extremely localized scattered light from a controlled 1-nm-scale plasmonic gap with unprecedented sensitivity. Whereas previous works have demonstrated individual aspects such as SNOM with plasmon-resonant tips (*55*–*57*), at LT (*30*, *58*, *59*), with QTF sensors (*60*–*61*), or in the FM mode (*62*), we integrate all those indispensable advances in ULA-SNOM to achieve high-resolution optical imaging. The combination of s-SNOM and picometer-scale plasmonics paves the way for the future advancement of single-molecule and atomic-scale optical microscopy.

## RESULTS

### ULA-SNOM configuration

**Fig. 1.**
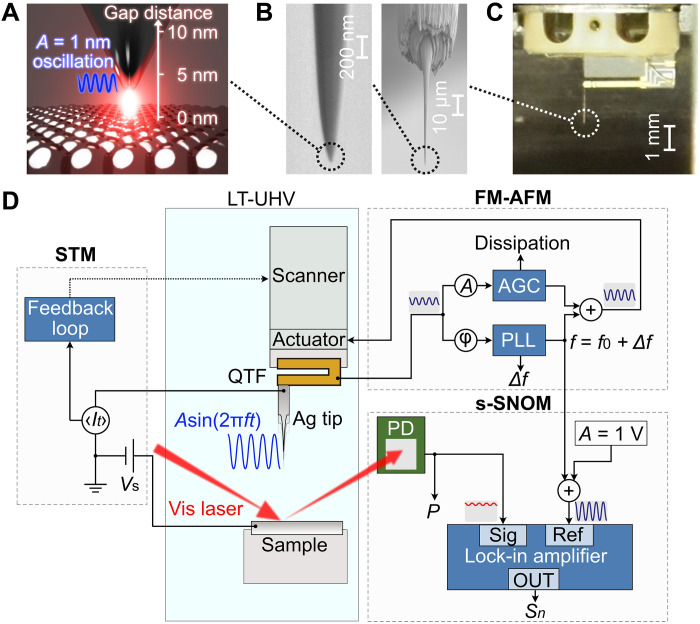
ULA-SNOM setup. (**A**) Schematic of ULA-SNOM. Light scattering from the highly confined picocavity-enhanced near-field can be detected by tip oscillation with an amplitude of 1 nm. (**B**) Scanning electron microscopy images of an Ag tip after the FIB polishing process. The left panel shows a magnified image of the tip apex of the image in the right panel. (**C**) Photo of a QTF sensor with the FIB-polished Ag tip mounted. (**D**) Circuit diagram of ULA-SNOM. The STM/FM-AFM unit is located in an UHV chamber at 8 K. A focused 633-nm laser beam illuminates the junction from outside the chamber and scattering light is collected by a PD outside the chamber. The PD signal is demodulated by a lock-in amplifier using the cantilever oscillation frequency f as a reference.

We performed ULA-SNOM by combining the laser illumination and light detection setup with a commercial LT-UHV STM/FM-AFM setup (Fig. 1; see also Materials and Methods). A continuous-wave visible laser beam enters the UHV chamber (wavelength λ=633 nm, incident power $P_{\text{inc}}=3–6$ mW, p-polarized) and is focused on the tip-sample junction through a lens mounted inside the STM/AFM unit at 8 K (fig. S1). The light scattered from the junction is collected by a second lens also installed inside the STM/AFM unit and is directed into a photodetector (PD) outside the UHV chamber to measure the power P of the scattered light. The apex of an electrochemically etched Ag tip is sharpened and polished by focused ion beam (FIB) milling (Fig. 1B) to obtain reproducible properties of the plasmonic nanocavity (*15*, *63*) and to reduce far-field scattered light from a rough tip shaft. The tip is mounted on a QTF sensor (Fig. 1C), which allows for the simultaneous detection of the STM tunneling current $I_{t}$ and the FM-AFM frequency shift Δf . When the cantilever is oscillated, the measured tunneling current is the time average over the oscillation cycle, denoted as $\langle I_{t}\rangle$ . The current signal is used for feedback control of the tip height z (Fig. 1D) as general in QTF-based STM/FM-AFM (*53*). The cantilever is resonantly oscillated with a frequency $f=f_{0}+\Delta f$ , which is fed back by an automatic gain controller to keep the oscillation amplitude A constant and by a phase-locked loop to obtain Δf induced by tip–sample interactions (*51*). We use a sine-wave output sin(2πft) from the oscillation controller as the reference signal for the lock-in detection in real time (Fig. 1D), obtaining n-th harmonic signals $S_{n}$ from the PD output. We verified the pure harmonic cantilever oscillation even at a tunneling regime (fig. S2), eliminating the possibility of artifacts in the $S_{n}$ signals due to higher harmonic components of the cantilever oscillation (*46*).

### Simultaneously recorded STM, FM-AFM, and s-SNOM signals

**Fig. 2.**
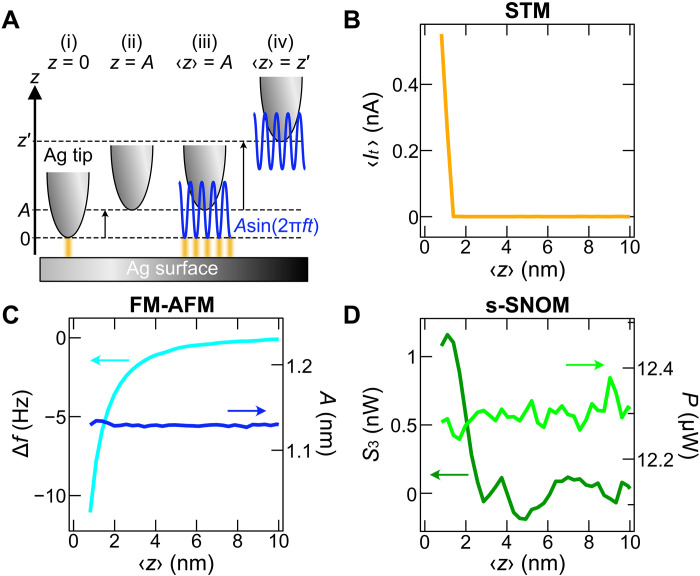
Approach curves of STM, FM-AFM, and s-SNOM. (**A**) Schematic side view of the way to obtain an approach curve with an oscillation amplitude A . The origin of z is defined as the tip height determined by the STM setpoint (sample bias $V_{s}=30$ mV and tunneling current $I_{t}=0.10$ nA) without cantilever oscillation, as depicted in (i). After the tip retraction by A (ii), the oscillation is started (iii). At the point, the tip height is described as z(t)=A[1+sin(2πft)] , where 〈z(t)〉=A and min[z(t)]=0 . Then, the tip is moved vertically to a given point 〈z〉=z′ (iv) to record an approach curve. The orange lines between the tip and sample depict tunneling current. (**B** to **D**) Approach curves of STM ( $\langle I_{t}\rangle$ ), FM-AFM ( A and Δf ), and s-SNOM ( P and $S_{3}$ ), respectively, recorded over an Ag(111) terrace ( $P_{\text{inc}}=3$ mW, A=1.13 nm).

The localization of the near-field light inside the junction can be characterized by measuring tip-approach curves, which represents the tip-height dependence of the scattered signal at a given oscillation amplitude A. Figure 2A shows the measurement procedure. First, the Ag tip is placed over an atomically flat Ag(111) surface without cantilever oscillation and with the STM feedback closed [defined as z=0 ; (i) in Fig. 2A]. Next we open the feedback loop and retract the tip from z=0 to z=A (ii). We then start the sinusoidal cantilever oscillation with an amplitude of A such that the tip oscillated around the center position z=A (iii). Therefore, the lowest tip height during the oscillation is equal to the setpoint distance, i.e., min[z(t)]=0 , ensuring that the tip does not crash into the surface when varying A . The instantaneous tip height during oscillation is expressed as z(t)=〈z〉+Asin(2πft) , where 〈z〉 is the time-averaged tip height equal to the center of oscillation. We then sweep the tip height to acquire approach curves in the range between 〈z〉=A and a given maximum height z′ [(iv) in Fig. 2A]. The approach curve of the s-SNOM signal $S_{n}\left(\langle z\rangle \right)$ is obtained along with the STM and FM-AFM signals.

Figure 2 (B to D) shows the STM signal ( $\langle I_{t}\rangle$ ), FM-AFM signals ( A and Δf ), and the scattered laser power P together with the third-harmonics s-SNOM signal $S_{3}$ , respectively, simultaneously recorded over a Ag terrace with a setpoint oscillation amplitude of 1.13 nm and during 633-nm laser illumination. Because a typical metal-tip–metal-sample gap distance during STM feedback is approximately 0.5 nm (*64*), we estimate the gap at z=1 nm to be ~1.5 nm. We obtained the curves at 〈z〉=1 to 150 nm. Only plots at 〈z〉≤10 nm are displayed in Fig. 2 because at larger tip heights, no signal exceeding the noise floor was observed in $\langle I_{t}\rangle$ , Δf , or $S_{3}$ (fig. S3). The time-averaged current $\langle I_{t}\rangle$ was detectable only at very close tip heights and shows a steep rise at 〈z〉<1.5 nm as expected from the exponential z dependence of tunneling current (Fig. 2B). The FM-AFM system always keeps A constant (Fig. 2C, right axis), whereas Δf decreases monotonically as 〈z〉 decreases (left axis) due to attractive forces between the Ag tip and Ag sample. These tip-height dependences are consistent with standard STM/FM-AFM operation (*65*), indicating that the laser illumination does not interfere the STM/FM-AFM performance.

As shown in the PD signal P (Fig. 2D, right axis), the near-field contribution to the total scattered power is faint and obscured by the noise of the PD channel, implying that P is dominated by background scattering with a constant intensity. However, lock-in detection at the third harmonic $S_{3}$ of the oscillation frequency (Fig. 2D, left axis) allows for the extraction of the near-field enhancement occurring only at very small gap distances. The steep increase of $S_{3}$ at 〈z〉<3 nm is consistent with the spatial confinement of electromagnetic fields in the tip-sample gaps (*5*, *17*, *19*). Notably, the STM, FM-AFM, and s-SNOM channels exhibit different tip-height thresholds for signals exceeding their noise floors ( 〈z〉≈1.5 nm for $\langle I_{t}\rangle$ , ~7 nm for Δf , and ~3 nm for $S_{3}$ ) as well as different steepness of the slopes, showing that their signals are independent and of different origins.

### Optimal oscillation amplitude for ULA-SNOM

**Fig. 3.**
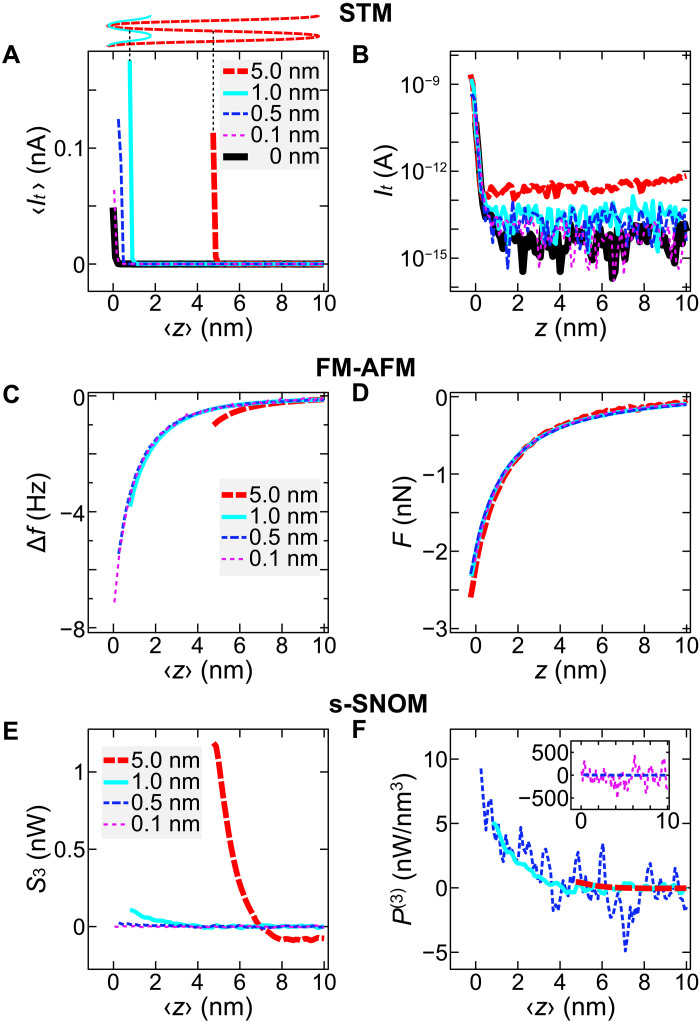
Amplitude-dependent approach curves and their converted/normalized curves. (**A**) Approach curves of $\langle I_{t}\rangle$ recorded over an Ag(111) terrace for different oscillation amplitudes A ( $P_{\text{inc}}=6$ mW). The sine waves on top of the graph illustrate the tip trajectories during the cantilever oscillation with *A* = 5.0 nm (red dotted curve) and 1.0 nm (cyan solid) at the minimum 〈z〉 points in the plots. (**B**) $I_{t}\left(z\right)$ curves converted from (A). (**C**) Approach curves of Δf simultaneously recorded with (A). (**D**) F(z) curves converted from (C). (**E**) Approach curves for $S_{3}$ simultaneously recorded with (A). (**F**) Normalized s-SNOM curves calculated from (E). The normalized curve with the lowest amplitude (*A* = 0.1 nm) is not shown in the main graph of (F) but in the inset (magenta dotted curve) because of the large noise. As a reference, the curve with *A* = 0.5 nm (blue dashed curves) is also shown in the inset. With any A , the origin of z is defined as the tip height determined by the STM setpoint at $V_{s}=30$ mV, $I_{t}=0.10$ nA without oscillation (see Fig. 2A).

As mentioned above, a small value of A is advantageous for detecting highly localized signals. However, if A is too small, this will reduce the signal-to-noise ratio in the lock-in detection. It is therefore important to examine the optimal oscillation amplitude to obtain the best localization at a reasonably high signal level. For this purpose, we measure approach curves with various values of A. Figure 3 (A, C, and E) shows the approach curves of $\langle I_{t}\rangle$ , Δf , and $S_{3}$ , respectively, recorded simultaneously over the Ag(111) surface for A between 0.1 and 5.0 nm. As a reference, $I_{t}\left(z\right)$ curve without cantilever oscillation is also shown (Fig. 3A, bold black curve). At the largest amplitude A=5.0 nm, all signals exceed their noise floors at much larger tip heights (for example, 〈z〉=5 nm for $\langle I_{t}\rangle$ ) compared to the plots at lower A values (see also Fig. 2 at A=1.13 nm). This behavior can be explained by tip trajectory during oscillation, as illustrated by the sine waves depicted on top of Fig. 3A. At the closest 〈z〉 , which depends on A as described above (Fig. 2A), the tip temporarily approaches the STM setpoint distance during each oscillation cycle, giving rise to signals in $\langle I_{t}\rangle$ , Δf , and $S_{3}$ . The approach curves of $\langle I_{t}\rangle$ and Δf can be converted into the instantaneous tunneling current $I_{t}$ (Fig. 3B) and vertical force F (Fig. 3D) at the bottom of the oscillation, i.e., z=min[z(t)] , using the Sader-Sugimoto (*66*) and Sader-Jarvis (*67*) formulae, respectively. The converted curves are consistent at any A , corroborating the stable, harmonic motion of the cantilever oscillation, which is critical to extract reliable s-SNOM signals upon demodulation (*46*). Notably, the conversions allow for the evaluation of the tunneling conductivity and interatomic force in narrow tip-sample gaps, e.g., at z=0 , using any A.

For s-SNOM, large A limits the information at narrow tip-sample gaps, unless sufficiently high harmonic channels are used (*32*, *42*). Here, we discuss the accessibility of s-SNOM signals in a small z range by normalizing the A-dependent intensity of the lock-in signals. The scattered light intensity P , which is modulated by the cantilever oscillation, can be expressed by a Taylor series as

$$\begin{array}{c} P\left[z\left(t\right)\right]=P\left[\langle z\rangle +A\text{sin}\left(2\pi ft\right)\right]\\ =\sum_{m=0}^{\infty }\frac{{\left(-A\right)}^{m}}{m!}P^{\left(m\right)}\left(\langle z\rangle \right){\text{sin}}^{m}\left(2\pi ft\right) \end{array}$$ (1)

where $P^{\left(m\right)}\left(\langle z\rangle \right)\equiv {\left(-1\right)}^{m}{\frac{d^{m}P}{dz^{m}}|}_{z=\langle z\rangle }$ . The lock-in signal corresponds to the time-averaged value of the input signal P multiplied with a sine-wave reference signal with a frequency of nf for the n-th harmonics, i.e.,

$$S_{n}\left(\langle z\rangle \right)=\langle P\left[z\left(t\right)\right]\text{sin}\left(2\text{π}\text{nft}+\phi_{n}\right)\rangle$$ (2)

where $\phi_{n}$ denotes the phase difference between the input and reference signals (see text S1). From Eqs. 1 and 2, $S_{n}$ is solved as

$$S_{n}\left(\langle z\rangle \right)=\frac{1}{n!2^{n}}A^{n}P^{\left(n\right)}\left(\langle z\rangle \right)+\sum_{i=1}^{\infty }c_{n+2i}A^{n+2i}P^{\left(n+2i\right)}\left(\langle z\rangle \right)$$ (3)

where $c_{n+2i}$ denotes the coefficient for the (n+2i)-th derivative component, which is much smaller than $\frac{1}{n!2^{n}}$ (see text S1). In the case of FM-AFM, A is constant at any z and a sufficiently low A eliminates the contribution of higher order terms. Therefore, the $S_{3}$ approach curve (Fig. 3E) can be normalized as $P^{\left(3\right)}\left(\langle z\rangle \right)=48A^{-3}S_{3}\left(\langle z\rangle \right)$ (Fig. 3F; see also fig. S4 for other n-th harmonics curves and their normalization).

After normalization, all approach curves recorded at different A yield the same exponential 〈z〉 dependence of the near-field signal (Fig. 3F), however, with different noise levels and minimum distances 〈z〉 . The largest amplitude ( A=5.0 nm) provides high signal-to-noise ratio, but it misses the information below 〈z〉≈5 nm and does not allow to extract the picocavity-enhanced near-field signal. Furthermore, at such a large A , the contribution of the higher order derivative components (see the second term on the right side of Eq. 3) to $S_{n}$ is no longer negligible, modifying the curve appearance from the original $P^{\left(n\right)}\left(\langle z\rangle \right)$ curve shape (see text S1). For these reasons, too large values of A are not suitable for the relatively low harmonics detection, such as n=1 to 4 . In contrast, at the smallest amplitude A=0.1 nm, no s-SNOM signal $S_{3}$ was measurable above the noise floor (inset in Fig. 3F). Its normalized curve has an approximately 50 times higher noise intensity than the signals detected at higher A . Therefore, we conclude that amplitudes of 0.5 to 1 nm are optimal for the ULA-SNOM experimental setup used. Note that this value depends on the intensity of light scattering from the near-field and the signal sensitivity of the light detection setup. Higher collection efficiency of the scattered light is expected to allow for s-SNOM signal detection with smaller A.

### s-SNOM imaging of Si monolayer islands on Ag(111)

**Fig. 4.**
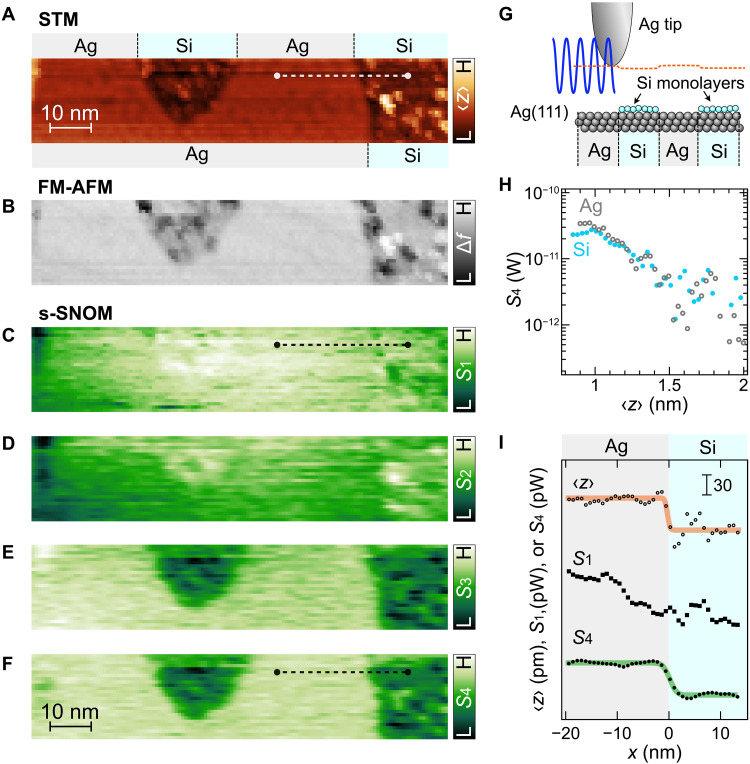
Simultaneously acquired STM, FM-AFM, and s-SNOM images. (**A**) STM topography, (**B**) FM-AFM Δf map, and (**C** to **F**) s-SNOM $S_{1}$ to $S_{4}$ maps simultaneously obtained (STM setpoint: $V_{s}=30$ mV, $\langle I_{t}\rangle =0.10$ nA, A = 1.0 nm; $P_{\text{inc}}=6$ mW). The highest (H) and lowest (L) values of each color bars are as follows: (H, L) = (0.32, −0.15) nm (A), (−0.31, −1.6) Hz (B), (21, 19) nW (C), (235, 19) pW (D), (151, 31) pW (E), and (51, −49) pW (F). (**G**) Side-view scheme of the atomic structures of the sampling area. Labels “Ag” and “Si” in (A) and (G) represent the bare Ag terrace and Si island on the terrace, respectively. (**H**) Approach curves of $S_{4}$ recorded over an Ag terrace (gray empty bullets) and an Si island on it (cyan filled bullets), recorded with another Ag tip (fig. S5H) than that for the maps in (A) to (F). The origin of 〈z〉 for both plots is defined by the STM setpoint over the Ag terrace (setpoint: $V_{s}=30$ mV and $I_{t}=0.10$ nA without oscillation). (**I**) Line profiles of the STM topographic height 〈z〉 (top), $S_{1}$ (middle), and $S_{4}$ (bottom) across an Ag-Si boundary on an identical terrace. The dotted lines in (A), (C), and (F) indicate the sampling position. The solid curves are the fitting curves for 〈z〉 (top) and $S_{4}$ (bottom) with error functions.

To demonstrate the lateral resolution and optical contrast of ULA-SNOM, we use an Ag(111) surface partially covered by ultrathin Si islands (Fig. 4A). According to the STM appearance, the islands, which partially cover the terraces and step edges (see fig. S5A for the overview STM image), are ascribed to amorphous Si films (*68*). Note that the islands appear darker in the image than the Ag terrace despite being located on the terrace. This contrast originates from the lower local density of states of Si compare to Ag, which competes with the topographic height difference. The coexistence of the Si monolayer islands and bare Ag surfaces minimizes the topographic height difference between Si and Ag (Fig. 4G), while retaining the plasmon enhancement effect between the Ag tip and Ag substrate (*69*).

We record simultaneous STM, FM-AFM, and s-SNOM images of the sample (Fig. 4, A to F). Both the QTF-based FM-AFM and the high harmonic lock-in detection require rather long acquisition times (~30 times slower than the scan speed in standard STM imaging mode), rendering imaging over larger areas at constant tip height challenging. Therefore, the tip height was controlled by the STM feedback loop to avoid thermal drift during the slow scan. As schematically shown in Fig. 4G, the scanned area has two Si islands partially covering a Ag terrace. Under the STM feedback, the Ag-tip–Ag-surface gaps are slightly narrower by ~50 pm than the Ag-Si gaps (the topmost line profile in Fig. 4I and the orange dotted curve in Fig. 4G). The FM-AFM Δf map (Fig. 4B) shows several dark spots inside the Si islands, presumably due to Si atoms/clusters in the Si monolayer attractively interacting with the Ag tip (*70*). On the other locations in the Si island, the Δf value is similar to that over Ag, and forces applied over Ag and Si are also comparable [see fig. S6 for the Δf(〈z〉) and F(z) curves]. The different appearance in each map suggests that simultaneous STM/FM-AFM/s-SNOM mapping provides complementary information on the scanned area due to the different signal origin of each image.

Figure 4 (C to F) shows s-SNOM images for different harmonics $S_{n}$ . The image appearance changes with n . The images of $S_{1}$ and $S_{2}$ (Fig. 4, E and F) are not sensitive to the presence of the Si islands. The middle plot of Fig. 4I shows the line profile of the $S_{1}$ map over a boundary between Ag and Si (dotted line in Fig. 4C), where the boundary gives no signal change. The Si island on the left side in the maps (Fig. 4, C and D) is imaged slightly brighter than the bare Ag, which is presumably attributed to the topographic artifact; the tip height over Si is slightly lower than Ag (Fig. 4G), providing the faint signal difference. The topographic effect on $S_{1}$ and $S_{2}$ was confirmed by mapping across an Ag step (see text S2). On the contrary, at higher harmonics $S_{3}$ (Fig. 4E) and $S_{4}$ (Fig. 4F), the image contrast changes as the Si islands now exhibit darker than the Ag terraces. This contrast is opposite of the topographic artifact effect, strongly indicating that the images of $S_{3}$ and $S_{4}$ are sensitive to changes in the local dielectric environment caused by the Si islands.

The n dependence of the s-SNOM appearance agrees with the common understanding that the localized near-field signal is predominantly detected at high n (*23*, *32*, *38*, *42*). The lower harmonics are not sensitive to the ultraconfined near-field in the 1-nm-scale gap. Hence, whereas the image appearances of $S_{1}$ and $S_{2}$ are dominated by topographic artifacts, the images of $S_{3}$ and $S_{4}$ show true optical, i.e., dielectric, material contrast. This is further corroborated by the observation that an atomic-scale structural change in the tip apex modifies the magnitude of the material contrast (see text S2 and fig. S5).

Figure 4H shows $S_{4}$ approach curves recorded over a Ag terrace and a Si island on it, also indicating that the $S_{4}$ signal over Si is smaller than that over Ag at small 〈z〉 . This trend is consistent with previous calculations (*24*, *38*) reporting that at 633 nm, Ag gives a larger scattering intensity in $S_{n}$ than Si due to the difference in the real part of the dielectric constant ( −18+i0.5 for Ag versus 15+i0.2 for Si). Considering that the image contrast originates only from a single atomic layer of Si, the contrast between the two surface regions is quite notable.

We note that our implementation of ULA-SNOM is based on a self-homodyne scheme (see fig. S1), which arises from the interference between tip-localized scattering and background scattering (*22*). In our s-SNOM imaging, nevertheless, the length scales of the cantilever oscillation amplitude, tip-sample gap distance, and scanned size are much smaller than the wavelength of the light ( A<λ/600 ), where the amplitude and phase of the background field should be constant. This in part is facilitated by the FIB-polished tip and the atomically flat single-crystalline surface under UHV conditions, which substantially reduce uncontrolled scattering. Furthermore, the aforementioned results of the n-dependent s-SNOM images (Fig. 4, C to F) and the consistency of the Ag/Si contrast with the numerical prediction (*24*, *38*) support that the background effect alone cannot account for the observed contrast. ULA-SNOM is, in principle, compatible with interferometric detection methods such as pseudo-heterodyne, which have been established to provide amplitude- and phase-resolved responses in a fully background-free manner (*22*, *71*). Combining ULA-SNOM with such interferometric techniques offers the potential for a full characterization of the dielectric function at the atomic scale.

Last, we estimate the lateral resolution of s-SNOM and compare it to that of STM by taking line profiles along the images across a material boundary. The bottommost plot in Fig. 4I shows the line profile of the $S_{4}$ signal recorded simultaneously with the STM topography (topmost plot). The faint oscillation spanning over a few nanometers in the STM line profile presumably originates from Friedel oscillations on Ag(111) (*72*). Both profiles exhibit step shapes at the boundary between Ag and Si, in contrast to the profile of $S_{1}$ without material contrast (middle plot in Fig. 4I). To quantitatively evaluate the lateral resolution, we conducted the peak fitting analysis of the line profiles, as in previous studies (*38*, *40*, *41*). We use an error function, $y\left(x\right)=y_{0}+c\;\text{erf}\left(\frac{x-x_{0}}{\sqrt{2}\sigma }\right)$ , where y(x) is the signal profile, $y_{0}$ and c are the offset and step-height coefficient, respectively, $x_{0}$ is the boundary position, and σ denotes the full width at half maximum. The fitting (solid curves in Fig. 4I) results in σ=0.39±0.26 for STM and 1.06±0.13 nm for s-SNOM $S_{4}$ . The observed difference in the spatial resolution between STM and s-SNOM provides further evidence that the contrast in the higher-harmonics s-SNOM images is not caused by topographic tip-height changes or tip motion (*73*) but reveals true optical contrast. The ability to resolve optical contrast with a spatial resolution as small as 1 nm in elastic light scattering provides an approach for optical surface analysis at the nearly atomic scale.

## DISCUSSION

We demonstrated the successful implementation and development of ULA-SNOM using light scattering from a stable plasmonic tip-sample nanojunction at a cryogenic temperature. We conducted FIB polishing of a plasmonic Ag tip mounted to the tuning fork sensor of FM-AFM and show that the highly confined near-field in the 1-nm-scale gap can be detected by demodulation and lock-in detection of the scattering signal at higher harmonics of the oscillation frequency of the QTF. We image the optical contrast between a bare Ag(111) surface and monoatomic Si islands on the surface with a lateral resolution of 1 nm using the fourth harmonics of the cantilever oscillation frequency. ULA-SNOM will be of interest for the optical characterization of a wide range of conducting and insulating nanomaterials exhibiting optical and dielectric heterogeneity at the atomic scale. The integration of s-SNOM in the LT-UHV STM/FM-AFM setup enables the simultaneous detection of multiple independent observables including electric conductivity (STM), interatomic attractive/repulsive forces (FM-AFM), and dielectric constant (s-SNOM), which promises new insights into, e.g., the photophysics of single defects and molecules and the optical properties of atomically sharp interfaces.

## MATERIALS AND METHODS

### ULA-SNOM setup

ULA-SNOM was performed with an LT-UHV STM/FM-AFM machine (CreaTec Fischer & Co. GmbH; base pressure < 5 × 10^−11^, a sample temperature of 8 K). The optical configuration on a self-homodyne scheme is depicted in fig. S1A. The output voltage of the Si-biased PD was converted into the light power P by the calibration with a Si-photodiode laser power meter. We confirmed that comparable results were obtained with another optical configuration of a back-scattering geometry (fig. S1, B to E). As a QTF sensor for the FM-AFM operation, we used a qPlus sensor (*52*) on which a Ag tip was mounted (CreaTec Fischer & Co. GmbH; spring constant of 1800 N/m, *Q* value of ~10^4^, sample-free resonance frequency $f_{0}$ of 18.8 kHz; Fig. 1C) and an oscillation controller (Nanonis OC4, SPECS Surface Nano Analysis GmbH). The sine-wave output with a frequency f from the controller was connected to the reference-signal input of a lock-in amplifier (HF2LI, Zurich Instruments), demodulating the PD signals (Fig. 1D). The quadrature (out-of-phase) component of each harmonics was always zero (see text S1), while each in-phase components were monitored as an s-SNOM channel.

### Tip fabrication

We sharpened and polished the apex of the electrochemically etched Ag tip mounted on the sensor (Fig. 1B) by Ga^+^ FIB milling. During the FIB process, the tunneling-current electrode of the sensor was grounded to prevent charging of the tip. Notably, while the FIB fabrication for STM tips have been reported (*15*, *63*, *74*), this is the first report for the FIB process of a tip mounted on a QTF sensor. After the sensor with the FIB-polished tip was introduced into the UHV chamber, the tip apex was further adjusted by mild poking into a clean Ag surface by the STM-based tip-height control to get a strong plasmon resonance (*15*).

### Sample fabrication

We used a single-crystalline Ag(111) surface (MaTeck GmbH) cleaned by multiple cycles of Ar^+^ sputtering and annealing. For the Si/Ag sample preparation (Fig. 4A), we used a home-made Si evaporator placed ~30 cm away from the cleaning stage for the Ag(111) surface in the UHV chamber. The evaporator has a direct-current-heated Si(111) plate (Siegert Wafer GmbH) flash annealed at 1200°C. During the Si evaporation, the cleaned Ag(111) sample was heated at 227° to 230°C and faced the evaporator.

## Supplementary Materials

### This PDF file includes:

Supplementary Text Figs. S1 to S6 References
