# Supplementary material for: Scattering near-field optical microscopy at 1-nm resolution using ultralow tip oscillation amplitudes
Source: Sci Adv. 2025 Jun 11;11(24):eadu1415. doi: 10.1126/sciadv.adu1415 (PMC12154227; doi:10.1126/sciadv.adu1415)
Supplement: Supplementary file 1 — Supplementary Text Figs. S1 to S6 References [file sciadv.adu1415_sm.pdf]

Science Advances
AAAS

Supplementary Materials for

Scattering near-field optical microscopy at 1-nm resolution using ultralow tip oscillation amplitudes

Akitoshi Shiotari *et al.*

Corresponding author: Akitoshi Shiotari, shiotari@fhi-berlin.mpg.de

Sci. Adv. **11**, eadu1415 (2025)
DOI: 10.1126/sciadv.adu1415

This PDF file includes:

Supplementary Text
Figs. S1 to S6
References

Supplementary Text

Supplementary Text 1: Normalization of s-SNOM approach curves

Here, we show the detail of the Taylor series for the scattered light intensity P modulated by the cantilever oscillation with an amplitude of A and a frequency of f (Eq. 1 in the main text);

$$\begin{aligned}
P[z(t)] &= P[\langle z\rangle + A\sin\theta] \\
&= P(\langle z\rangle) \\
&\quad + A\left.\frac{\mathrm{d}P}{\mathrm{d}z}\right|_{z=\langle z\rangle}\sin\theta \\
&\quad + \frac{1}{2}A^2\left.\frac{\mathrm{d}^2P}{\mathrm{d}z^2}\right|_{z=\langle z\rangle}\sin^2\theta \\
&\quad + \frac{1}{6}A^3\left.\frac{\mathrm{d}^3P}{\mathrm{d}z^3}\right|_{z=\langle z\rangle}\sin^3\theta \\
&\quad + \frac{1}{24}A^4\left.\frac{\mathrm{d}^4P}{\mathrm{d}z^4}\right|_{z=\langle z\rangle}\sin^4\theta \\
&\quad + \frac{1}{120}A^5\left.\frac{\mathrm{d}^5P}{\mathrm{d}z^5}\right|_{z=\langle z\rangle}\sin^5\theta \\
&\quad + \frac{1}{720}A^6\left.\frac{\mathrm{d}^6P}{\mathrm{d}z^6}\right|_{z=\langle z\rangle}\sin^6\theta + \cdots ,
\end{aligned} \tag{S1}$$

where $\theta \equiv 2\pi f t$.

As shown in the main text, we define $(-1)^n \left.\frac{\mathrm{d}^nP}{\mathrm{d}z^n}\right|_{z=\langle z\rangle}$ as $P^{(n)}(\langle z\rangle)$. This definition including coefficient $(-1)^n$ is compatible with the typical display style of SNOM approach curves $S_n(\langle z\rangle)$; when the scattering light increases exponentially as the tip approaches (i.e., $\mathrm{d}z < 0$), the corresponding $P^{(n)}(\langle z\rangle)$ represent exponential curves similar to $P(z)$, i.e., $P^{(n)}(\langle z\rangle) > 0$ at low z.

Using multiple-angle formulas, $\sin^m\theta$ can be expanded as follows;

$$
\begin{aligned}
P[z(t)] &= P(\langle z\rangle) \\
&\quad -AP^{(1)}(\langle z\rangle)\sin\theta \\
&\quad +\frac{1}{4}A^2P^{(2)}(\langle z\rangle)\,(1-\cos 2\theta) \\
&\quad -\frac{1}{24}A^3P^{(3)}(\langle z\rangle)\,(3\sin\theta-\sin 3\theta) \\
&\quad +\frac{1}{192}A^4P^{(4)}(\langle z\rangle)\,(3-4\cos 2\theta+\cos 4\theta) \\
&\quad -\frac{1}{1920}A^5P^{(5)}(\langle z\rangle)\,(10\sin\theta-5\sin 3\theta+\sin 5\theta) \\
&\quad +\frac{1}{23040}A^6P^{(6)}(\langle z\rangle)\,(10-15\cos 2\theta+6\cos 4\theta-\cos 6\theta)+\cdots\,. \qquad \text{(S2)}
\end{aligned}
$$

As described in Eq. 2 of the main text, the lock-in signal corresponds to

$$S_n(\langle z\rangle) = \langle P[z(t)]\sin(n\theta+\phi_n)\rangle \qquad \text{(S3)}$$

for the n-th harmonics. Among the components of $\sin(m\theta)$ or $\cos(m\theta)$ in Eq. S2, only the component at $m=n$ gives a non-zero time-averaged value, as $\langle\sin^2 n\theta\rangle = \langle\cos^2 n\theta\rangle = \frac{1}{2}$. For example, the first harmonic signal S_1 is maximized at $\phi_1=\pi$ as

$$
\begin{aligned}
S_1(\langle z\rangle) &= \langle P[z(t)](-\sin\theta)\rangle \\
&= AP^{(1)}(\langle z\rangle)\left\langle\sin^2\theta\right\rangle \\
&\quad +\frac{1}{24}A^3P^{(3)}(\langle z\rangle)\left\langle 3\sin^2\theta\right\rangle \\
&\quad +\frac{1}{1920}A^5P^{(5)}(\langle z\rangle)\left\langle 10\sin^2\theta\right\rangle+\ldots \\
&= \frac{1}{2}AP^{(1)}(\langle z\rangle)+\frac{3}{48}A^3P^{(3)}(\langle z\rangle)+\frac{1}{384}A^5P^{(5)}(\langle z\rangle)+\cdots\,. \qquad \text{(S4)}
\end{aligned}
$$

In a similar manner, each lock-in signal is maximized at $\phi_n = \frac{n+1}{2}\pi$ as

$$
\begin{aligned}
S_2(\langle z\rangle) &= \frac{1}{8}A^2P^{(2)}(\langle z\rangle)+\frac{1}{96}A^4P^{(4)}(\langle z\rangle)+\frac{1}{3072}A^6P^{(6)}(\langle z\rangle)+\cdots\,, \qquad \text{(S5)}\\
S_3(\langle z\rangle) &= \frac{1}{48}A^3P^{(3)}(\langle z\rangle)+\frac{1}{768}A^5P^{(5)}(\langle z\rangle)+\cdots\,, \qquad \text{(S6)}\\
S_4(\langle z\rangle) &= \frac{1}{384}A^4P^{(4)}(\langle z\rangle)+\frac{1}{7680}A^6P^{(6)}(\langle z\rangle)+\cdots\,. \qquad \text{(S7)}
\end{aligned}
$$

Equation 3 in the main text corresponds to a simplified description of Equations S4 to S7. Note that practically, depending on the lock-in amplifier, the experimental phase values ϕ_n can shift from

the theoretical values. Assuming that $P^{(5)}$, $P^{(6)}$, and higher order derivatives are negligibly small, the n-th derivative of the scattering laser power P with respect to $\langle z \rangle$ is described using the lock-in signals S_n as follows:

$$P^{(1)}(\langle z \rangle) \approx \frac{2}{A}\left[S_1(\langle z \rangle) - 3S_3(\langle z \rangle)\right], \tag{S8}$$

$$P^{(2)}(\langle z \rangle) \approx \frac{8}{A^2}\left[S_2(\langle z \rangle) - 4S_4(\langle z \rangle)\right], \tag{S9}$$

$$P^{(3)}(\langle z \rangle) \approx \frac{48}{A^3} S_3(\langle z \rangle), \tag{S10}$$

$$P^{(4)}(\langle z \rangle) \approx \frac{384}{A^4} S_4(\langle z \rangle). \tag{S11}$$

Figures S4A, D, G, and J show the approach curves of S_1 to S_4, respectively, recorded with several oscillation amplitudes $A = 0.1$–5.0 nm. Using different A provides different curve appearances, but the curves can be matched by the normalization. Figures S4B, E, H, and K show the $P^{(1)}(\langle z \rangle)$ to $P^{(4)}(\langle z \rangle)$ curves calculated from Eqs. S8 to S11, respectively. Each n-th derivative value is consistent with any A used. On the one hand, small A causes severe noise at higher n due to the coefficient of $1/A^n$ in the equations. For example, in the third (fourth) derivative channel, the normalized curve(s) with $A = 0.1$ nm ($A = 0.5$ and 0.1 nm) is (are) too noisy to read the signal appearance like an exponential function [Figs. S4H and J (K and L)]. On the other hand, with a too large A, higher order harmonics components is no longer negligible in S_n signals. For example, as shown in Eq. S4, the S_1 curve has the contribution of $A^3 P^{(3)}(\langle z \rangle)$ in addition to the major factor of $AP^{(1)}(\langle z \rangle)$. As a result, the raw approach curves with $A = 5.0$ nm (Figs. S4A and D) appear to deviate substantially from the curves with the other smaller A. Figure S4C (S4F) show the first (second) derivative curve reproduced by only considering S_1 (S_2) signals, i.e., assuming that $\mathrm{d}^n P/\mathrm{d}z^n \approx n!(-2/A)^n S_n$. In this rough approximation, the calculated curve with $A = 5.0$ nm does not match those with the other A (Figs. S4C and F). In contrast, as described above, the more accurate approximation using Eqs. S8 and S9 improves the matching (Figs. S4B and E). Note that the calculated $P^{(1)}$ curve with $A = 5.0$ nm still deviates slightly from the other curves, possibly due to the contribution of higher harmonics, i.e., $A^5 P^{(5)}$ in Eq. S4. Therefore, from the dataset, $A = 1.0$ nm is the optimal condition under which all the S_1 to S_4 channels well reproduce the n-th differential curves with a reasonable signal-to-noise ratio. Note that as mentioned in the main text, the optimal amplitude value is expected to depend on the experimental setup used, such as the tip

apex structure and the efficiency of the light collection.

Supplementary Text 2: ULA-SNOM mapping over a Ag atomic step

The Ag(111) surface partially covered by ultrathin Si islands was first characterized by overview STM imaging without cantilever oscillation (Fig. S5A). The appearance is in good agreement with the previous STM study of amorphous Si films on Ag(111) (*68*).

In addition to the Si islands on an identical Ag terrace (Fig. 4 in the main text), we obtained ULA-SNOM maps including a Ag step. As schematically shown in Fig. S5B, the scanned area has two Ag terraces separated by a single Ag-atomic step, both of which are partially covered by Si islands. The bare Ag terraces (regions I and III), Si islands (II and IV), and the monoatomic Ag step (between II and III) are easily identified by the contrast in the STM image (Fig. S5C). The appearances of the four regions are useful to understand the contrast in each image. Regions I and II have the same Ag terrace height, whereas regions I and III have the same Ag surface but with a different height by one Ag atomic layer, and regions I and IV have the same atom-layer numbers with different atomic species (Ag and Si) in the topmost layer. While the STM feedback ensures a constant tip–sample gap distance on the same material (e.g., regions I and III), the Ag-tip–Ag-surface gaps are slightly narrower by ~50 pm than the Ag–Si gaps (orange dotted curves in Fig. S5B).

The images of S_1 and S_2 (Figs. S5E and S5F) are not sensitive to the presence of the Si islands, as shown in Figs. 4C and 4D in the main text; however, the images show a contrast with regions I and II (III and IV) apparently darker (brighter), similar to the contrast-inverted surface topography. The topography-synchronized contrast in S_1 and S_2 is probably caused by the adjustment of the tip–sample distances at the two terraces across the Ag step (Fig. S5B) and scattering contributions from less localized fields.

At higher harmonics S_3 and S_4 (Figs. S5G and S5H), the image contrast changes as the Si islands in regions II and IV now exhibit darker than the Ag terraces in regions I and III, which is consistent with the mapping results over an identical Ag terrace (Figs. 4E and 4F in the main text). The high resolution s-SNOM mapping enables us for optical surface analysis in the ∼1-nm scale. For example, based on the contrast of the S_4 image, the bright spot with a diameter of ∼2 nm (marked by the dotted circles in Figs. S5C and S5H) is not composed of Si, although it is located

on an Si island, but can rather be assigned to an Ag cluster.

On the maps, we also observed the effect of an atomic-scale structural change in the tip on the images. We observed an accidental tip change during the scan (black arrows in Figs. S5C to S5H), as evidenced by a sudden jump of the simultaneously recorded STM and FM-AFM signals. Because the same islands and the Ag step were observed similarly even after the change, this is attributed to a change in the atomic structure of the tip apex. Notably, this minor tip change has a notable impact on the contrast in S_3 and S_4, but only a minor effect on S_1. This indicates that the atomic structure of the Ag tip apex tunes the plasmonic field in the tip–sample gap, leading to the modification of the optical contrast in the s-SNOM maps. Such atomic-level structures potentially forms plasmonic picocavities (*19, 20*), which also contributed to the plasmonic field intensity and confinement in the tip–sample junction. Revealing the existence of picocavities and utilizing them would further improve the spatial resolution of ULA-SNOM.

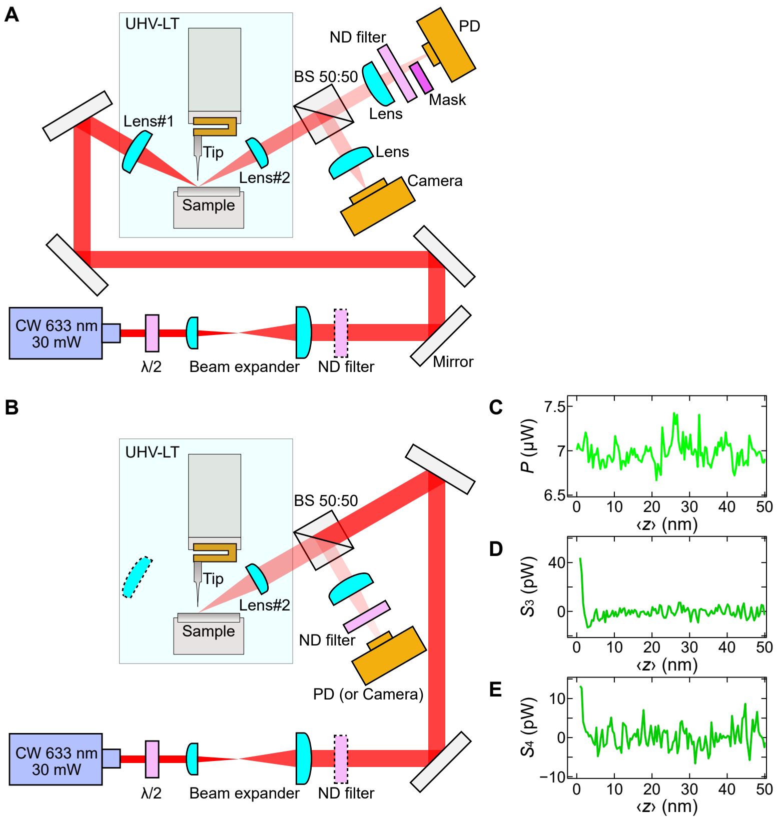

Figure S1: **Two optical configurations for ULA-SNOM.** (**A**) Configuration we mainly used. Continuous-wave 633-nm laser output entered into the UHV chamber and was focused into the tip–sample junction via Lens #1 (NA = 0.4) mounted in the STM/FM-AFM unit. The scattering light from the junction was collected by Lens #2 (NA = 0.2) together with reflecting light and was directed outside the chamber to a PD through an neutral density (ND) filter and a mask. To adjust the scattering light intensity to a PD-detectable range, the mask was used for blocking the strong light directly reflecting by the sample independent of the tip existence. (**B**) Another optical configuration of a back-scattering geometry. Lens #2 was used both for incident light focusing and for scattering light collection. The scattering light was directed to the PD via a beam splitter (BS). (**C** to **E**) Approach curves of P, S_3, and S_4, respectively, recorded over a bare Ag(111) terrace with the configuration in (B). The high harmonic s-SNOM channels have signals in a very close tip–sample gap, which is in good agreement with the results with the configuration in (A). This verifies that both configurations are capable of ULA-SNOM measurements. Nevertheless, the s-SNOM signals in the latter configuration (B) are generally weaker than those in the former (A) because in the latter configuration, the incident laser intensity is reduced by passing through the BS.

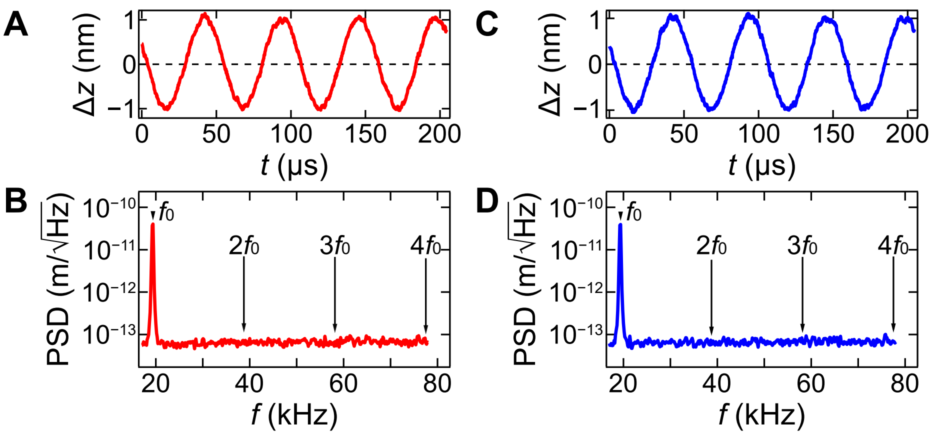

Figure S2: **Monitoring the cantilever oscillation of the QTF sensor with an oscilloscope and spectrum analyzer.** (**A**) Time trace of the cantilever oscillation $\Delta z(t)$ and (**B**) its power spectral density (PSD). The Ag tip was far from more than 350 nm from the Ag(111) surface. The cantilever oscillation was excited by a piezo actuator with the resonance frequency, *i.e.*, $f = f_0$, and the oscillation amplitude A was set at 1.0 nm. (**C**) Time trace and (**D**) PSD after the tip was approached to the sample. The tip height was controlled by the STM feedback with sample bias V_s = 30 mV, time-averaged tunneling current $\langle I_t \rangle$ = 0.10 nA with the cantilever oscillation, which is the same set-point as that for the s-SNOM maps shown in Fig. 4 of the main text. Because the frequency shift Δf at the tip height is much smaller than f_0 ($|\Delta f / f_0| < 0.04\%$), the position of the resonance peak in the spectrum appears unchanged from that in (B). Both before and after the tip approach, the oscillation shows pure harmonicity without detectable higher-harmonics components ($2f_0$, $3f_0$, and $4f_0$), as indicated in (B) and (D). This verifies that the lock-in signals of the scattering light (Fig. 2) and their difference in the maps (Fig. 4) at low tip heights do not originate from anharmonic cantilever motion but the near-field. This result is in constant to anharmonic oscillation of a Si cantilever with tapping-mode AFM at low tip heights (*46, 49*). We note that the detection of higher harmonic components of the cantilever oscillation with a QTF sensor was reported (*75*); however, the sensitive detection is owed to special properties of the sensor (the enhancement of the piezoelectric current output at high frequencies) (*53, 76*). The amplitudes of higher harmonics are intrinsically very small (~0.1 pm for A = 1 nm) (*76*) due to the stiffness of the cantilever (a spring constant of 1800 N/m, two orders of magnitude larger than Si cantilevers).

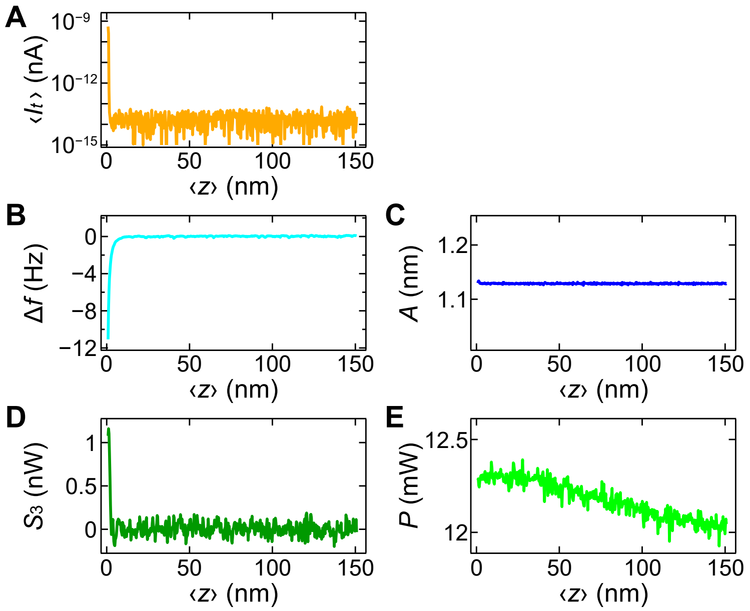

Figure S3: **The same approach curves as those shown in Figs. 2B to 2D of the main text, but with the full tip-height range recorded.** (**A**) $\langle I_t \rangle$, (**B**) Δf, (**C**) A, (**D**) S_3, and (**E**) P.

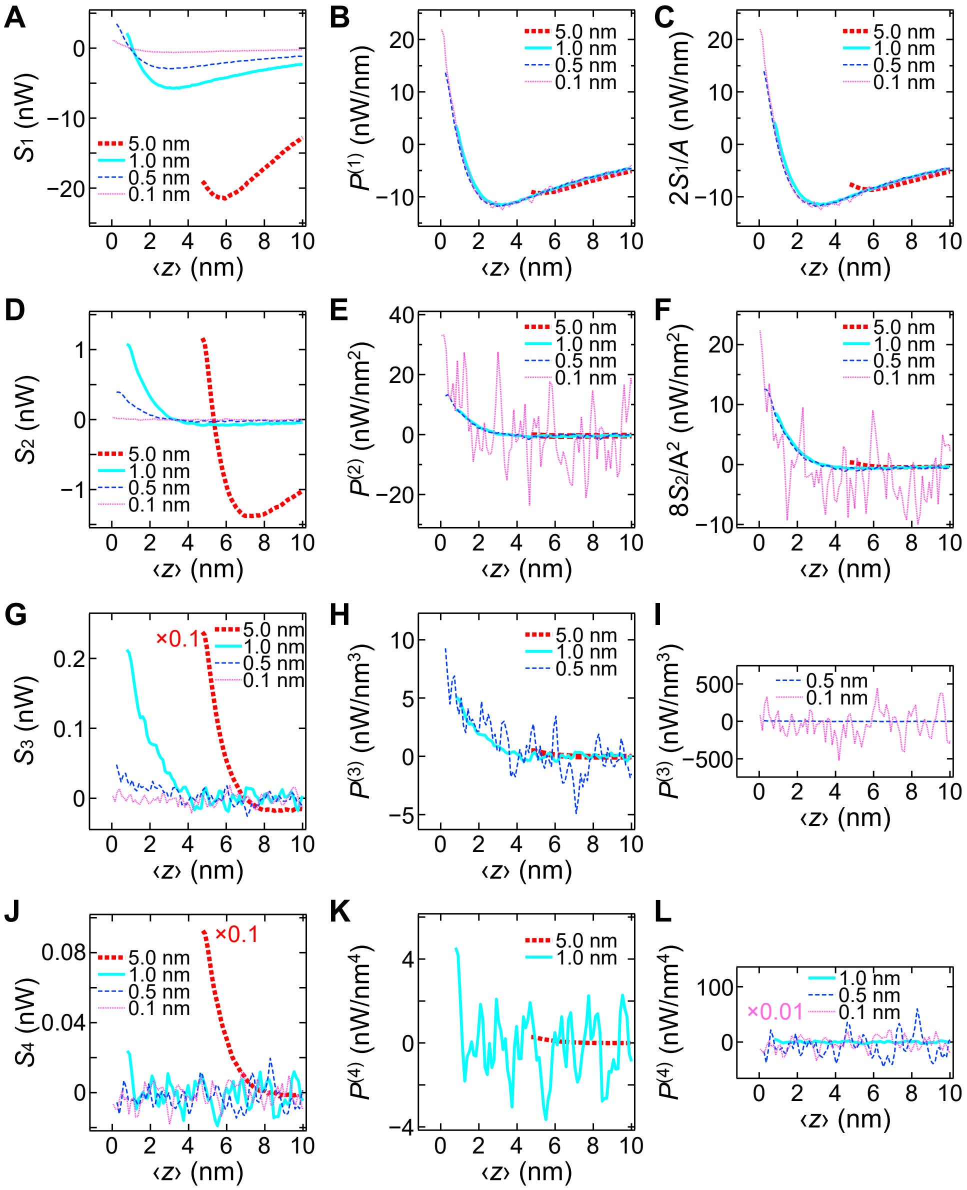

Figure S4: **Approach curves of S_n with different A and their normalized curves.** (**A**) S_1, (**B**) $P^{(1)}$ calculated from S_1 and S_3, and (**C**) $P^{(1)}$ calculated only from S_1. (**D**) S_2, (**E**) $P^{(2)}$ calculated from S_2 and S_4, and (**F**) $P^{(2)}$ calculated only from S_2. (**G**) S_3, (**H**) $P^{(3)}$ calculated from S_3 with larger A, and (**I**) $P^{(3)}$ calculated from S_3 with lower A. (**J**) S_4, (**K**) $P^{(4)}$ calculated from S_4 with larger A, and (**L**) $P^{(4)}$ calculated from S_4 with lower A. The plots in (G) to (I) are the same as those shown in Figs. 3E and 3F of the main text.

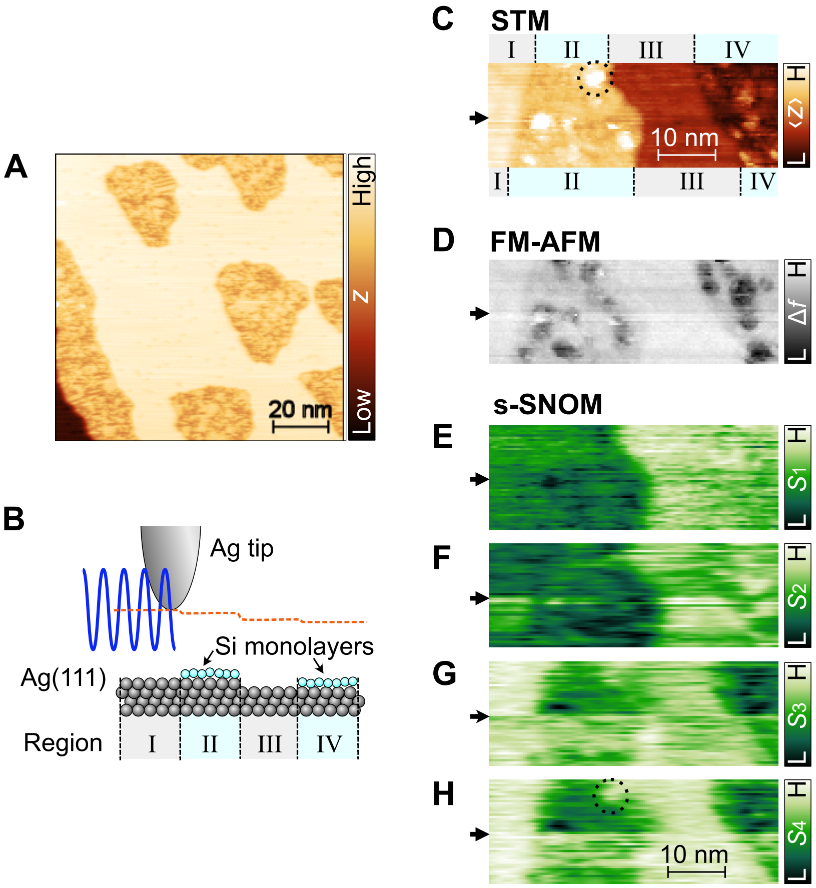

Figure S5: **Simultaneously acquired STM, FM-AFM, and s-SNOM images of a different area from that for Figs. 4A to 4F in the main text.** (**A**) Typical STM image of amorphous Si monolayer islands on Ag(111) obtained with the FIB-polished Ag tip without laser illumination (set-point: V_s = 1 V and I_t = 0.1 nA without cantilever oscillation). (**B**) Side-view scheme of the atomic structures of the sampling area. Regions I–IV correspond to the bare Ag on the upper terrace, Si island on the upper terrace, bare Ag on the lower terrace, and Si island on the lower terrace, respectively. The orange dotted line schematically indicates the tip-height trajectory. (**C**) STM topography, (**D**) FM-AFM Δf map, and (**E** to **H**) s-SNOM S_1 to S_4 maps simultaneously obtained with laser illumination (STM set-point: V_s = 30 mV, $\langle I_t \rangle$ = 0.10 nA, A = 1.0 nm; P_{inc} = 6 mW). The arrows indicate the position when the tip-apex structure accidentally changed. The black dotted circles in (C) and (H) mark a nanocluster in region II.

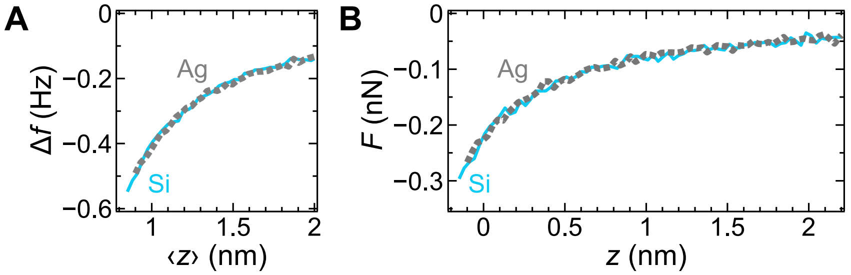

Figure S6: **Frequency shift and force curves simultaneously recorded with s-SNOM approach curves** (**A**) $\Delta f(\langle z \rangle)$ recorded over Ag (dotted gray curve) and Si (solid cyan curve). Each curve was simultaneously obtained with the s-SNOM approach curve $S_4(\langle z \rangle)$ shown in Fig. 4H of the main text. The origin of $\langle z \rangle$ for both curves is defined by the STM set-point over the Ag terrace, as well as that in Fig. 4H. (**B**) $F(z)$ curves converted from the curves in (A). While the S_4 intensity on Si differs from that on Ag at small $\langle z \rangle$ (Fig. 4H), Δf and F recording over the two locations are comparable at any tip heights.

REFERENCES AND NOTES

1. J. F. Schultz, S. Li, S. Jiang, N. Jiang, Optical scanning tunneling microscopy based chemical imaging and spectroscopy. *J. Phys. Condens. Matter* **32**, 463001 (2020).

2. R. Gutzler, M. Garg, C. R. Ast, K. Kuhnke, K. Kern, Light–matter interaction at atomic scales. *Nat. Rev. Phys.* **3**, 441–453 (2021).

3. M. Müller, Imaging surfaces at the space–time limit: New perspectives of time-resolved scanning tunneling microscopy for ultrafast surface science. *Prog. Surf. Sci.* **99**, 100727 (2023).

4. H. Lee, D. Y. Lee, M. G. Kang, Y. Koo, T. Kim, K.-D. Park, *Tip-enhanced photoluminescence nano-spectroscopy and nano-imaging.*, *Nanophotonics* **9**, 3089–3110 (2020).

5. X. Wang, S.-C. Huang, S. Hu, S. Yan, B. Ren, Fundamental understanding and applications of plasmon-enhanced Raman spectroscopy. *Nat. Rev. Phys.* **2**, 253–271 (2020).

6. R. Zhang, Y. Zhang, Z. C. Dong, S. Jiang, C. Zhang, L. G. Chen, L. Zhang, Y. Liao, J. Aizpurua, Y. Luo, J. L. Yang, J. G. Hou, Chemical mapping of a single molecule by plasmon-enhanced Raman scattering. *Nature* **498**, 82–86 (2013).

7. J. Lee, K. T. Crampton, N. Tallarida, V. A. Apkarian, Visualizing vibrational normal modes of a single molecule with atomically confined light. *Nature* **568**, 78–82 (2019).

8. X. H. Qiu, G. V. Nazin, W. Ho, Vibrationally resolved fluorescence excited with submolecular precision. *Science* **299**, 542–546 (2003).

9. K. Kuhnke, C. Große, P. Merino, K. Kern, Atomic-scale imaging and spectroscopy of electroluminescence at molecular interfaces. *Chem. Rev.* **117**, 5174–5222 (2017).

10. B. Yang, G. Chen, A. Ghafoor, Y. Zhang, Y. Zhang, Y. Zhang, Y. Luo, J. Yang, V. Sandoghdar, J. Aizpurua, Z. Dong, J. G. Hou, Sub-nanometre resolution in single-molecule photoluminescence imaging. *Nat. Photonics* **14**, 693–699 (2020).

11. H. Imada, M. Imai-Imada, K. Miwa, H. Yamane, T. Iwasa, Y. Tanaka, N. Toriumi, K. Kimura, N. Yokoshi, A. Muranaka, M. Uchiyama, T. Taketsugu, Y. K. Kato, H. Ishihara, Y. Kim, Single-molecule laser nanospectroscopy with micro–electron volt energy resolution. *Science* **373**, 95–98 (2021).

12. E. Kazuma, J. Jung, H. Ueba, M. Trenary, Y. Kim, STM studies of photochemistry and plasmon chemistry on metal surfaces. *Prog. Surf. Sci.* **93**, 163–176 (2018).

13. X. Zhu, Y. Xu, C. Zhao, C. Jia, X. Guo, Recent advances in photochemical reactions on single-molecule electrical platforms. *Macromol. Rapid Commun.* **43**, e2200017 (2022).

14. A. Rosławska, K. Kaiser, M. Romeo, E. Devaux, F. Scheurer, S. Berciaud, T. Neuman, G. Schull, Submolecular-scale control of phototautomerization. *Nat. Nanotechnol.* **19**, 738–743 (2024).

15. Y. Park, I. Hamada, A. Hammud, T. Kumagai, M. Wolf, A. Shiotari, Atomic-precision control of plasmon-induced single-molecule switching in a metal–semiconductor nanojunction. *Nat. Commun.* **15**, 6709 (2024).

16. M. Imai-Imada, H. Imada, K. Miwa, Y. Tanaka, K. Kimura, I. Zoh, R. B. Jaculbia, H. Yoshino, A. Muranaka, M. Uchiyama, Y. Kim, Orbital-resolved visualization of single-molecule photocurrent channels. *Nature* **603**, 829–834 (2022).

17. P. G. Etchegoin, E. Le Ru, A perspective on single molecule SERS: Current status and future challenges. *Phys. Chem. Chem. Phys.* **10**, 6079–6089 (2008).

18. C. Zhang, B.-Q. Chen, Z.-Y. Li, Optical origin of subnanometer resolution in tip-enhanced Raman mapping. *J. Phys. Chem. C* **119**, 11858–11871 (2015).

19. M. Barbry, P. Koval, F. Marchesin, R. Esteban, A. G. Borisov, J. Aizpurua, D. Sánchez-Portal, Atomistic near-field nanoplasmonics: reaching atomic-scale resolution in nanooptics. *Nano Lett.* **15**, 3410–3419 (2015).

20. F. Benz, M. K. Schmidt, A. Dreismann, R. Chikkaraddy, Y. Zhang, A. Demetriadou, C. Carnegie, H. Ohadi, B. De Nijs, R. Esteban, J. Aizpurua, J. J. Baumberg, Single-molecule optomechanics in "picocavities". *Science* **354**, 726–729 (2016).

21. R. Hillenbrand, B. Knoll, F. Keilmann, Pure optical contrast in scattering-type scanning near-field microscopy. *J. Microsc.* **202**, 77–83 (2001).

22. X. Chen, D. Hu, R. Mescall, G. You, D. N. Basov, Q. Dai, M. Liu, Modern scattering-type scanning near-field optical microscopy for advanced material research. *Adv. Mater.* **31**, e1804774 (2019).

23. B. Knoll, F. Keilmann, Enhanced dielectric contrast in scattering-type scanning near-field optical microscopy. *Opt. Commun.* **182**, 321–328 (2000).

24. M. B. Raschke, C. Lienau, Apertureless near-field optical microscopy: Tip–sample coupling in elastic light scattering. *Appl. Phys. Lett.* **83**, 5089–5091 (2003).

25. J. Chen, M. Badioli, P. Alonso-González, S. Thongrattanasiri, F. Huth, J. Osmond, M. Spasenović, A. Centeno, A. Pesquera, P. Godignon, A. Z. Elorza, N. Camara, F. J. García de Abajo, R. Hillenbrand, F. H. L. Koppens, Optical nano-imaging of gate-tunable graphene plasmons. *Nature* **487**, 77–81 (2012).

26. Z. Fei, A. S. Rodin, G. O. Andreev, W. Bao, A. S. McLeod, M. Wagner, L. M. Zhang, Z. Zhao, M. Thiemens, G. Dominguez, M. M. Fogler, A. H. Castro Neto, C. N. Lau, F. Keilmann, D. N. Basov, Gate-tuning of graphene plasmons revealed by infrared nano-imaging. *Nature* **487**, 82–85 (2012).

27. D. N. Basov, M. M. Fogler, F. J. García de Abajo, Polaritons in van der Waals materials. *Science* **354**, aag1992 (2016).

28. T. Low, A. Chaves, J. D. Caldwell, A. Kumar, N. X. Fang, P. Avouris, T. F. Heinz, F. Guinea, L. Martin-Moreno, F. Koppens, Polaritons in layered two-dimensional materials. *Nat. Mater.* **16**, 182–194 (2017).

29. M. M. Qazilbash, M. Brehm, B.-G. Chae, P.-C. Ho, G. O. Andreev, B.-J. Kim, S. J. Yun, A. V. Balatsky, M. B. Maple, F. Keilmann, H.-T. Kim, D. N. Basov, Mott transition in VO_2 revealed by infrared spectroscopy and nano-imaging. *Science* **318**, 1750–1753 (2007).

30. A. S. McLeod, E. Van Heumen, J. G. Ramirez, S. Wang, T. Saerbeck, S. Guenon, M. Goldflam, L. Anderegg, P. Kelly, A. Mueller, M. K. Liu, I. K. Schuller, D. N. Basov, Nanotextured phase coexistence in the correlated insulator V_2O_3. *Nat. Phys.* **13**, 80–86 (2017).

31. I. Amenabar, S. Poly, W. Nuansing, E. H. Hubrich, A. A. Govyadinov, F. Huth, R. Krutokhvostov, L. Zhang, M. Knez, J. Heberle, A. M. Bittner, R. Hillenbrand, Structural analysis and mapping of individual protein complexes by infrared nanospectroscopy. *Nat. Commun.* **4**, 2890 (2013).

32. J. Nishida, A. Otomo, T. Koitaya, A. Shiotari, T. Minato, R. Iino, T. Kumagai, Sub-tip-radius near-field interactions in nano-FTIR vibrational spectroscopy on single proteins. *Nano Lett.* **24**, 836–843 (2024).

33. R. Jacob, S. Winnerl, M. Fehrenbacher, J. Bhattacharyya, H. Schneider, M. T. Wenzel, H.-G. von Ribbeck, L. M. Eng, P. Atkinson, O. G. Schmidt, M. Helm, Intersublevel spectroscopy on single InAs-quantum dots by terahertz near-field microscopy. *Nano Lett.* **12**, 4336–4340 (2012).

34. M. Eisele, T. L. Cocker, M. A. Huber, M. Plankl, L. Viti, D. Ercolani, L. Sorba, M. S. Vitiello, R. Huber, Ultrafast multi-terahertz nano-spectroscopy with sub-cycle temporal resolution. *Nat. Photonics* **8**, 841–845 (2014).

35. M. Zizlsperger, S. Nerreter, Q. Yuan, K. B. Lohmann, F. Sandner, F. Schiegl, C. Meineke, Y. A. Gerasimenko, L. M. Herz, T. Siday, M. A. Huber, M. B. Johnston, R. Huber, In situ nanoscopy of single-grain nanomorphology and ultrafast carrier dynamics in metal halide perovskites. *Nat. Photonics* **18**, 975–981 (2024).

36. F. Zenhausern, Y. Martin, H. K. Wickramasinghe, Scanning interferometric apertureless microscopy: Optical imaging at 10 Angstrom resolution. *Science* **269**, 1083–1085 (1995).

37. J. Koglin, U. C. Fischer, H. Fuchs, Material contrast in scanning near-field optical microscopy at 1–10 nm resolution. *Phys. Rev. B* **55**, 7977–7984 (1997).

38. R. Hillenbrand, F. Keilmann, Material-specific mapping of metal/semiconductor/dielectric nanosystems at 10 nm resolution by backscattering near-field optical microscopy. *Appl. Phys. Lett.* **80**, 25–27 (2002).

39. F. Huth, A. Govyadinov, S. Amarie, W. Nuansing, F. Keilmann, R. Hillenbrand, Nano-FTIR absorption spectroscopy of molecular fingerprints at 20 nm spatial resolution. *Nano Lett.* **12**, 3973–3978 (2012).

40. K.-T. Lin, S. Komiyama, Y. Kajihara, Tip size dependence of passive near-field microscopy. *Opt. Lett.* **41**, 484–487 (2016).

41. S. Mastel, A. A. Govyadinov, C. Maissen, A. Chuvilin, A. Berger, R. Hillenbrand, Understanding the image contrast of material boundaries in IR nanoscopy reaching 5 nm spatial resolution. *ACS Photonics* **5**, 3372–3378 (2018).

42. F. Wang, S. Yang, S. Li, S. Zhao, B. Cheng, C. Xia, High resolution and high signal-to-noise ratio imaging with near-field high-order optical signals. *Nano Res.* **15**, 8345–8350 (2022).

43. E. Betzig, R. J. Chichester, Single molecules observed by near-field scanning optical microscopy. *Science* **262**, 1422–1425 (1993).

44. Z. Zafar, S. Yi, J. Li, C. Li, Y. Zhu, A. Zada, W. Yao, Z. Liu, X. Yue, Recent development in defects engineered photocatalysts: An overview of the experimental and theoretical strategies. *Energy Environ. Mater.* **5**, 68–114 (2022).

45. R. Krutokhvostov, A. A. Govyadinov, J. M. Stiegler, F. Huth, A. Chuvilin, P. S. Carney, R. Hillenbrand, Enhanced resolution in subsurface near-field optical microscopy. *Opt. Exp.* **20**, 593–600 (2012).

46. R. Esteban, R. Vogelgesang, K. Kern, Full simulations of the apertureless scanning near field optical microscopy signal: Achievable resolution and contrast. *Opt. Exp.* **17**, 2518–2529 (2009).

47. F. Mooshammer, M. A. Huber, F. Sandner, M. Plankl, M. Zizlsperger, R. Huber, Quantifying nanoscale electromagnetic fields in near-field microscopy by Fourier demodulation analysis. *ACS Photonics* **7**, 344–351 (2020).

48. A. Mannoni, F. Quercioli, B. Tiribilli, C. Ascoli, P. Baschieri, C. Frediani, Measuring topography and refractive index of channel waveguides with a hybrid AFM-SNOM. *J. Light. Technol.* **16**, 388–394 (1998).

49. R. Hillenbrand, M. Stark, R. Guckenberger, Higher-harmonics generation in tapping-mode atomic-force microscopy: Insights into the tip–sample interaction. *Appl. Phys. Lett.* **76**, 3478–3480 (2000).

50. A. Bek, R. Vogelgesang, K. Kern, Optical nonlinearity versus mechanical anharmonicity contrast in dynamic mode apertureless scanning near-field optical microscopy. *Appl. Phys. Lett.* **87**, 163115 (2005).

51. T. R. Albrecht, P. Grütter, D. Horne, D. Rugar, Frequency modulation detection using high-Q cantilevers for enhanced force microscope sensitivity. *J. Appl. Phys.* **69**, 668–673 (1991).

52. F. J. Giessibl, High-speed force sensor for force microscopy and profilometry utilizing a quartz tuning fork. *Appl. Phys. Lett.* **73**, 3956–3958 (1998).

53. F. J. Giessibl, The qPlus sensor, a powerful core for the atomic force microscope. *Rev. Sci. Instrum.* **90**, 011101 (2019).

54. T. Siday, J. Hayes, F. Schiegl, F. Sandner, P. Menden, V. Bergbauer, M. Zizlsperger, S. Nerreter, S. Lingl, J. Repp, J. Wilhelm, M. A. Huber, Y. A. Gerasimenko, R. Huber, All-optical subcycle microscopy on atomic length scales. *Nature* **629**, 329–334 (2024).

55. U. C. Fischer, D. W. Pohl, Observation of single-particle plasmons by near-field optical microscopy. *Phys. Rev. Lett.* **62**, 458–461 (1989).

56. F. Huth, A. Chuvilin, M. Schnell, I. Amenabar, R. Krutokhvostov, S. Lopatin, R. Hillenbrand, Resonant antenna probes for tip-enhanced infrared near-field microscopy. *Nano Lett.* **13**, 1065–1072 (2013).

57. R.-H. Jiang, C. Chen, D.-Z. Lin, H.-C. Chou, J.-Y. Chu, T.-J. Yen, Near-field plasmonic probe with super resolution and high throughput and signal-to-noise ratio. *Nano Lett.* **18**, 881–885 (2018).

58. J. Döring, H.-G. von Ribbeck, M. Fehrenbacher, S. C. Kehr, L. M. Eng, Near-field resonance shifts of ferroelectric barium titanate domains upon low-temperature phase transition. *Appl. Phys. Lett.* **105**, 053109 (2014).

59. M. Dapolito, X. Chen, C. Li, M. Tsuneto, S. Zhang, X. Du, M. Liu, A. Gozar, Scattering-type scanning near-field optical microscopy with Akiyama piezo-probes. *Appl. Phys. Lett.* **120**, 013104 (2022).

60. A. Naber, H.-J. Maas, K. Razavi, U. C. Fischer, Dynamic force distance control suited to various probes for scanning near-field optical microscopy. *Rev. Sci. Instrum.* **70**, 3955–3961 (1999).

61. P. G. Gucciardi, G. Bachelier, A. Mlayah, M. Allegrini, Interferometric measurement of the tip oscillation amplitude in apertureless near-field optical microscopy. *Rev. Sci. Instrum.* **76**, 036105 (2005).

62. N. Satoh, K. Kobayashi, K. Matsushige, H. Yamada, Near-field light detection of a photo induced force by atomic force microscopy with frequency modulation. *Jpn. J. Appl. Phys.* **56**, 08LB03 (2017).

63. S. Liu, M. Müller, Y. Sun, I. Hamada, A. Hammud, M. Wolf, T. Kumagai, Resolving the correlation between tip-enhanced resonance Raman scattering and local electronic states with 1 nm resolution. *Nano Lett.* **19**, 5725–5731 (2019).

64. Y.-h. Zhang, P. Wahl, K. Kern, Quantum point contact microscopy. *Nano Lett.* **11**, 3838–3843 (2011).

65. M. Ternes, C. González, C. P. Lutz, P. Hapala, F. J. Giessibl, P. Jelínek, A. J. Heinrich, Interplay of conductance, force, and structural change in metallic point contacts. *Phys. Rev. Lett.* **106**, 016802 (2011).

66. J. E. Sader, Y. Sugimoto, Accurate formula for conversion of tunneling current in dynamic atomic force spectroscopy. *Appl. Phys. Lett.* **97**, 043502 (2010).

67. J. E. Sader, S. P. Jarvis, Accurate formulas for interaction force and energy in frequency modulation force spectroscopy. *Appl. Phys. Lett.* **84**, 1801–1803 (2004).

68. D. Solonenko, O. D. Gordan, G. Le Lay, D. R. T. Zahn, P. Vogt, Comprehensive Raman study of epitaxial silicene-related phases on Ag(111). *Beilstein J. Nanotechnol.* **8**, 1357–1365 (2017).

69. J. F. Schultz, N. Jiang, Characterizations of two-dimensional materials with cryogenic ultrahigh vacuum near-field optical microscopy in the visible range. *J. Vac. Sci. Technol. A* **40**, 040801 (2022).

70. Z. Majzik, M. R. Tchalala, M. Švec, P. Hapala, H. Enriquez, A. Kara, A. J. Mayne, G. Dujardin, P. Jelínek, H. Oughaddou, Combined AFM and STM measurements of a silicene sheet grown on the Ag(111) surface. *J. Phys. Condens. Matter* **25**, 225301 (2013).

71. N. Ocelic, A. Huber, R. Hillenbrand, Pseudoheterodyne detection for background-free near-field spectroscopy. *Appl. Phys. Lett.* **89**, 101124 (2006).

72. Y. Hasegawa, P. Avouris, Direct observation of standing wave formation at surface steps using scanning tunneling spectroscopy. *Phys. Rev. Lett.* **71**, 1071–1074 (1993).

73. B. Hecht, H. Bielefeldt, Y. Inouye, D. W. Pohl, L. Novotny, Facts and artifacts in near-field optical microscopy. *J. Appl. Phys.* **81**, 2492–2498 (1997).

74. H. Böckmann, M. Müller, A. Hammud, M.-G. Willinger, M. Pszona, J. Waluk, M. Wolf, T. Kumagai, Near-field spectral response of optically excited scanning tunneling microscope junctions probed by single-molecule action spectroscopy. *J. Phys. Chem. Lett.* **10**, 2068–2074 (2019).

75. S. Hembacher, F. J. Giessibl, J. Mannhart, Force microscopy with light-atom probes. *Science* **305**, 380–383 (2004).

76. F. J. Giessibl, Higher-harmonic atomic force microscopy. *Surf. Interface Anal.* **38**, 1696–1701 (2006).
